# Tetracycline ameliorates silica-induced pulmonary inflammation and fibrosis via inhibition of caspase-1

**DOI:** 10.1186/s12931-022-01937-7

**Published:** 2022-02-07

**Authors:** Konrad Peukert, Folkert Steinhagen, Mario Fox, Caroline Feuerborn, Susanne Schulz, Benjamin Seeliger, Patrick Schuss, Matthias Schneider, Stilla Frede, Andrea Sauer, Christian Putensen, Eicke Latz, Christoph Wilhelm, Christian Bode

**Affiliations:** 1 Department of Anesthesiology and Intensive Care Medicine, University Hospital Bonn, Venusberg-Campus 1, 53127 Bonn, Germany; 2 Department of Anesthesiology and Intensive Care Medicine, SHG Hospital Voelklingen, Richard-Str. 5-9, 66333 Voelklingen, Germany; 3 Department of Respiratory Medicine and German Centre of Lung Research (DZL), Hannover Medical School, Carl-Neuberg-Str. 1, 30635 Hannover, Germany; 4 Department of Neurosurgery, University Hospital Bonn, Venusberg-Campus 1, 53127 Bonn, Germany; 5 Institute of Innate Immunity, University Hospital Bonn, Venusberg-Campus 1, 53127 Bonn, Germany; 6 Institute of Clinical Chemistry and Clinical Pharmacology, University Hospital Bonn, Venusberg-Campus 1, 53127 Bonn, Germany

**Keywords:** Anti-bacterial agents, Immunomodulation, Inflammasomes, Pyroptosis, Silicosis, Lung injury, NLR Proteins, Silicon dioxide

## Abstract

**Background:**

Inhalation of dust containing silica particles is associated with severe pulmonary inflammation and lung injury leading to chronic silicosis including fibrotic remodeling of the lung. Silicosis represents a major global health problem causing more than 45.000 deaths per year. The inflammasome-caspase-1 pathway contributes to the development of silica-induced inflammation and fibrosis via IL-1β and IL-18 production. Recent studies indicate that tetracycline can be used to treat inflammatory diseases mediated by IL-1β and IL-18. Therefore, we hypothesized that tetracycline reduces silica-induced lung injury and lung fibrosis resulting from chronic silicosis via limiting IL-1β and IL-18 driven inflammation.

**Methods:**

To investigate whether tetracycline is a therapeutic option to block inflammasome-caspase-1 driven inflammation in silicosis, we incubated macrophages with silica alone or combined with tetracycline. The in vivo effect of tetracycline was determined after intratracheal administration of silica into the mouse lung.

**Results:**

Tetracycline selectively blocks IL-1β production and pyroptotic cell death via inhibition of caspase-1 in macrophages exposed to silica particles. Consistent, treatment of silica-instilled mice with tetracycline significantly reduced pulmonary caspase-1 activation as well as IL-1β and IL-18 production, thereby ameliorating pulmonary inflammation and lung injury. Furthermore, prolonged tetracycline administration in a model of chronic silicosis reduced lung damage and fibrotic remodeling.

**Conclusions:**

These findings suggest that tetracycline inhibits caspase-1-dependent production of IL-1β in response to silica in vitro and in vivo. The results were consistent with tetracycline reducing silica-induced pulmonary inflammation and chronic silicosis in terms of lung injury and fibrosis. Thus, tetracycline could be effective in the treatment of patients with silicosis as well as other diseases involving silicotic inflammation.

## Background

Silicosis is a pulmonary disease caused by inhalation of silica particles in occupational or environmental settings. With more than 45.000 deaths per year globally, silicosis represents one of the major occupational diseases worldwide [1, 2]. Inhaled silica particles, encountered in occupations including mining and construction, accumulate in small airways and alveoli inaccessible for mucocilial clearance [3, 4]. Ingestion of silica particles by alveolar macrophages leads to acute pulmonary inflammation hallmarked by excessive production of inflammatory mediators and cell death [4]. Subsequently, ingested silica particles are released and re-ingested by other macrophages, amplifying a vicious circle of inflammation and cell death [4]. Dependent on the time being exposed to silica particles, silicosis can be subdivided into an acute, inflammatory form marked by silicoproteinosis and a chronic form characterized by pulmonic collagen deposition and fibrotic remodeling of the lung [2, 5, 6]. To embank morbidity and mortality associated with irreversible progressive and incurable silicosis, there is an urgent need for new therapies preventing prolonged inflammation and collagen deposition in silicosis [2].

Increasing evidence highlights the proinflammatory cytokines IL-1β and IL-18 as key drivers in the development of silicosis [3–5]. IL-1β and IL-18 production is regulated via the inflammasome-caspase-1 pathway. Inflammasomes are multiprotein complexes consisting of a sensor e.g. the nucleotide-binding oligomerization domain–like receptor (NLR) family, e.g. pyrin domain–containing 3 (NLRP3), the adapter protein apoptosis-associated speck-like protein containing a CARD domain (ASC) and caspase-1 [7]. Assembly of the inflammasome complex and subsequent caspase-1 activation requires two signals. Signal 1 comprises the activation of pattern-recognition receptors (PRR) including Toll-like receptors (TLRs) by pathogen-associated molecular patterns (PAMPs) such as lipopolysaccharides (LPS). Subsequently nuclear factor kappa-light-chain-enhancer of activated B cells (NF-ƙB) initiates transcription of inflammasome components including pro-caspase-1, pro–IL-1β and pro-IL-18 [7]. The source of signal 1 in macrophages during silicosis is not conclusively identified and may be mediated by locally produced cytokines or respiratory infections [3]. Silica particles function as signal 2 leading to the activation of the sensor such as NLRP3 and subsequent assembly of the inflammasome complex [3, 4]. This activates caspase-1 that results in proteolytic activation of pro-IL-1β and pro-IL-18 into bio-active IL-1β and IL-18. Further caspase-1 facilitates pyroptosis, a highly inflammatory form of cell death characterized by rupture of the cell membrane and distinct LDH release [3, 4, 7].

Tetracycline and its derivatives have been evaluated in studies of inflammatory diseases where they are reported to be both safe and have immunomodulatory activity [8–12]. Several experimental and clinical studies highlighted anti-inflammatory and lung protective effects of tetracycline derivatives in inflammatory lung diseases including idiopathic pulmonary fibrosis and cystic fibrosis [13–19]. Recent evidence suggests that tetracycline limits both cytokine production of IL-1β and IL-18 as well as pyroptosis via selective inhibition of caspase-1, thereby reducing acute lung injury [20]. Therefore, we hypothesized that tetracycline reduces silica-induced lung injury via limiting caspase-1 driven inflammation. As silicosis is a chronic disease, we further explored the long-term effects of tetracycline following repeated exposure to silica.

## Material and methods

### Mice

Wild type (wt) mice C57BL/6 J, 8–10 weeks, male, (Charles River Laboratories, Sulzfeld, Germany) were kept in a pathogen-free facility. The animals were handled according to the principles of laboratory animal care (NIH publication No. 85–23, revised 1996). The animal procedures were in accordance with German legal guidelines and were approved by the responsible local authority for animal care (animal protocols: AZ 81-02.04.2018.A110).

### Silica preparation

Silica crystals (MIN-U-SIL-15) (mean particle length 5 μm) were purchased from US Silica (Berkeley Springs, WV) and used in all experiments. To avoid potential endotoxin contamination silica crystals were prepared as described previously [6].

### In vivo model

Mice were anesthetized by isoflurane (Pirmal, Mumbai, India) inhalation and intubated with a 20-gauge catheter. Silica (0.33 mg/mouse in 50 μl sterile PBS) or 50 μl sterile PBS (Thermo Fisher Scientific, Darmstadt, Germany) was instilled intratrachealy (i.t.) on days 0, 7, 14 and 21. Instantaneous mice were treated by intraperitoneal (i.p.) injection (75 μg/g mouse in 200 μl sterile PBS) of tetracycline (Sigma-Aldrich, St. Louis, MO, USA) or 200 μl sterile PBS and thereafter every 24 h for 10d. Afterwards i.p. injections were performed 3 times a week. Mice were sacrificed 24 h and 12 weeks after instillation. Bronchoalveolar lavage fluid (BALF) was collected from mice by twice instilling and removing 1 ml of PBS using a 20-gauge catheter. IL-1ß, IL-18 and albumin levels were analyzed by ELISA (R&D Systems, Minneapolis, MN, USA and Bethyl, Montgomery, TX, USA) and total protein concentration by BCA (Thermo Fisher Scientific). Single cell suspension was prepared and blocked with CD16/CD32 antibody (Ab) (2.4G2, BD Bioscience, Franklin Lakes, NJ, USA) to avoid non-specific binding of immunoglobulin to the Fc receptors. Dead cells were excluded by using LIVE/DEAD Fixable Dead Cell Stain kit (Thermo Fisher). Absolute cell numbers were determined by adding CaliBRITE APC-beads (BD Bioscience). FACS Canto II (BD Bioscience, Franklin Lakes, NJ, USA) (FACSDiva software 6.1.2), FlowJo software 10.6.1 (TreeStar, Ashland, OR, USA) and antibodies (Abs) against, CD45 (30-E11, eFluor450), Ly-6C (HK1.4, PE), F4/80 (BM8, PE-Cy7) (Thermo Fisher Scientific), CD3 (17A2 FITC eBiosience) and Ly-6G (148, APC) (BioLegend) were used for neutrophil, macrophage and lymphocyte characterization.

### Histopathologic analyses

Lungs were inflated (with a pressure of 15 cm H_2_O) and fixed with zinc-formalin (Z-fix; Anatech, Battle Creek, MI, USA). Lungs were embedded in paraffin, sectioned at 3 µm and stained with hematoxylin and eosin (H&E) (Sigma-Aldrich) or sirius red (Sigma-Aldrich) as described previously [5]. Two blinded investigators evaluated the samples according to a semi quantitative lung injury score [21].

### In vitro analyses

Bone marrow from wt mice was harvested. Bone marrow derived macrophages (BMDM) were differentiated for 4d in DMEM (Thermo Fisher Scientific) supplemented with 10% heat inactivated FCS (Biochrom, Berlin, Germany) and 10 ng/ml mouse macrophage colony-stimulating factor (m-CSF) (ImmunoTools, Friesoythe, Germany). Afterwards medium was removed, cells were scraped and seeded at 1 × 10^6^/ml in 24-well plates for 24 h before replacing medium and conducting experiments. Cells were primed with LPS for 1 h and afterwards stimulated with silica and co-incubated with tetracycline or VX765 (25 µM, specific caspase-1 inhibitor) (Invivogen) for 3 h. Supernatants were analyzed for, IL-1ß and TNF-α secretion by ELISA (R&D Systems). Pyroptosis was measured in cell supernatants via determination of LDH release by the CytoTox 96 assay (Promega, Madison, WI, USA).

### Immunoblotting

Cells were lysed in RIPA buffer (R&D Systems) containing protease inhibitors (Sigma-Aldrich) and total protein was determined by BCA (Thermo Fisher Scientific). Lysates or supernatants were separated by SDS-PAGE (NuPAGE, Thermo Fisher Scientific) and blotted onto nitrocellulose, nytrane membranes (GE healthcare, Chicago, IL, USA). Anti-mouse caspase-1, full-length and activated p20 fragment (mAb Casper-1, Adipogen Life Sciences, Liestal, Switzerland), ASC (anti-Asc, pAb (AL177), Adipogen Life Sciences), NLRP3 (mAB Cryo-2, Adipogen Life Sciences), IL-1 ß (anti-mIL-1β R&D Systems) were used as primary and horseradish-peroxidase-conjugated anti-rabbit and anti-mouse IgG (both Cell Signaling Technology, Beverly, MA, USA) as secondary antibodies. Chemiluminescent substrate (Biozym Scientific GmbH, Hessisch Oldendorf, Germany) was used for visualization.

### RT-PCR

Total RNA was extracted by TRIzol reagent (Invitrogen), as specified by the manufacturer; cDNA was synthesized with the cDNA Reverse Transcription kit (Applied Biosystems) and Real-Time (RT) PCR was performed as previously described [22]. Gene expression levels (normalized to 18 s) were calculated using the 2 (-DeltaDeltaC(T)) method. All reagents and probes used were purchased from Applied Biosystems (Darmstadt, Germany).

### SIRCOL assay

The level of collagen in the lung tissue was determined using the SIRCOL collagen assay (Biocolor LTD., UK) according to manufacturer’s instructions. Briefly, right lung lobes were removed, homogenized and collagen was solved in 0.5 M acetic acid and incubated with Sirius red dye. The absorbance was analyzed at 540 nm using a spectrophotometer revealing the amount of collagen in the lung.

### Statistics

Statistical analysis was performed using GraphPad Prism 8 Software (La Jolla, CA, USA). In nonparametric data variables were compared by Mann–Whitney U test. For more than two groups, overall group differences were assessed by Kruskal–Wallis test and intergroup-differences were assessed by ranksum-testing adjusting for multiple comparison by false discovery rate (Benjamini, Krieger and Yekutieli). Values of p < 0.05 were considered significant. All data are expressed as median with interquartile range.

## Results

### Tetracycline selectively inhibits silica induced IL-1ß production and pyroptosis

**Fig. 1:**
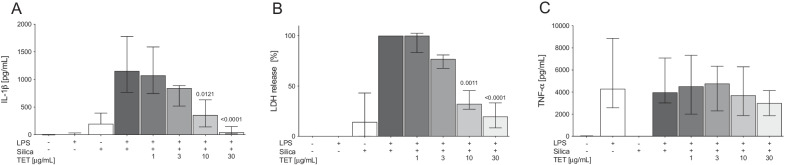
Tetracycline inhibits silica induced IL-1β production and pyroptosis. Murine BMDM were stimulated with either LPS (30 ng/ml) alone or in combination with silica (SIL, 1 mg/ml) and then treated with increasing doses of tetracycline (TET). IL-1ß (**A**), LDH **B** and TNF-α **C** concentrations were measured in supernatants with ELISA or LDH-Assay. Median with interquartile range of ≥ 4 independent experiments. Ranksum-testing adjusting for multiple comparison after Kruskal–Wallis test

To investigate the inhibitory effect of tetracycline on silica-induced activation of the inflammasome-caspase-1 pathway, we primed murine BMDM with LPS and stimulated the cells with silica in the presence or absence of tetracycline. As expected, silica exposure led to a significant release of IL-1ß in LPS primed BMDM (Fig. 1A). Tetracycline dose-dependently inhibited this IL-1ß production (p ≤ 0.0121) and pyroptosis associated LDH release (p ≤ 0.0011) (Fig. 1A, B). Consistent with previous results [20], inflammasome-caspase-1 independent TNF-α production was not affected by tetracycline (Fig. 1C). These results indicate that tetracycline selectively blocks silica-induced IL-1ß production and pyroptosis of BMDM.

### Tetracycline inhibits silica-induced activation of caspase-1

**Fig. 2:**
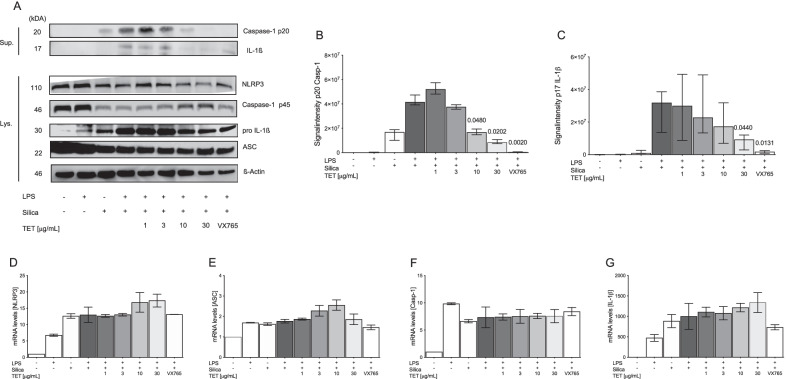
Tetracycline inhibits activation of caspase-1. Murine BMDM were stimulated as described in Fig. 1. Immunoblots of lysates (LY; caspase-1 (p45), ASC, pro-IL-1ß, NLRP3 and ß-Actin) and supernatants (SN; caspase-1 (p20) and mature IL-1ß) of wt BMDM (**A**). Representative blots from ≥ 3 independent experiments. Quantification of signal intensity of caspas-1 (p20) **B** and mature IL-1ß (**C**). NLRP3 (**B**), ASC (**C**), Casp-1 **D** and IL-1ß **E** mRNA levels were determined by qPCR using 18 s RNA as an endogenous control 3 independent experiments. Median with interquartile range of ≥ 3 independent experiments. Ranksum-testing adjusting for multiple comparison after Kruskal–Wallis test

Since tetracycline decreased both silica-induced IL-1ß production and pyroptosis, we conducted immunoblot analysis to examine whether tetracycline inhibits caspase-1 activation in BMDM in response to silica. Tetracycline dose-dependently reduced caspase-1 activation, indicated by less cleavage of the p45 caspase-1 precursor into its p20 subunit while expression of NLRP3 and ASC, which is upstream of caspase-1, was not affected (Fig. 2A, B). Consistent with the previous findings (Fig. 1A), active IL-1ß but not pro- IL-1ß was dose-dependently reduced by tetracycline (Fig. 2A, C). Of note, VX765, a selective caspase-1 inhibitor, served as a control in these experiments. To further evaluate potential effects of tetracycline on upstream TLR4-NF-ƙB signaling, expression of NF-ƙB-dependent genes encoding components of the inflammasome-caspase-1 pathway were investigated. Tetracycline had no effect on the expression of mRNA levels of NLRP3, ASC, pro-caspase-1 or pro-IL-1ß after stimulation with LPS and silica (Fig. 2D–G). These data suggest that tetracycline reduces silica-induced IL-1ß production by direct inhibition of caspase-1.

### Tetracycline inhibits pulmonary inflammation and caspase-1 in acute silicosis

**Fig. 3:**
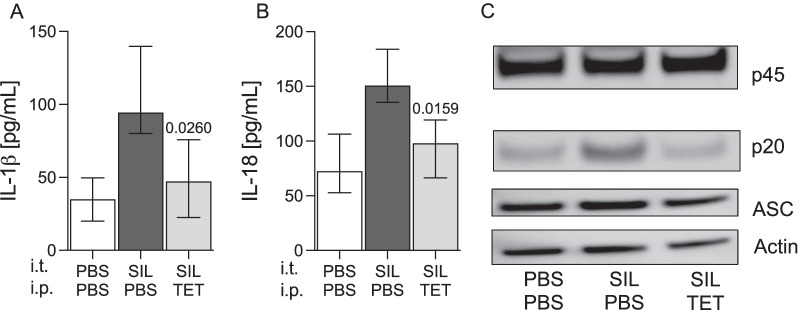
Tetracycline inhibits caspase-1 in silica exposed mice. C57BL/6 J mice were challenged i.t. with silica (0.33 mg/mouse) on day 0 and immediately afterwards treated with tetracycline (TET) (75 µg/g BW) or PBS i.p. 24 h after silica (SIL) exposure the concentration of IL-1ß **A** and IL-18 **B** in bronchoalveolar lavage fluid was determined by ELISA. Median with interquartile range of 3 independent experiments (SIL + TET (n = 6) vs. SIL + PBS (n = 6)) (PBS + PBS n = 6), Mann–Whitney Test

To test whether tetracycline ameliorates caspase-1 dependent pulmonary inflammation in acute silicosis, we challenged C57Bl/6 J mice i.t. with silica particles and treated them with tetracycline. 24 h after silica instillation, IL-1ß and IL-18 concentrations were analyzed in BALF. As shown in Fig. 3A, B, silica exposure markedly induced pulmonary IL-1ß and IL-18 levels. Treatment with tetracycline significantly inhibited IL-1ß (p = 0.0260) and IL-18 (p = 0.0159) production (Fig. 3A, B). We next examined activation of caspase-1 in lung homogenates by immunoblotting. In comparison to PBS treated controls, silica induced the activation of caspase-1, which was indicated by increased p20 fragment. In contrast, tetracycline inhibited caspase-1 activation in this model and cleavage of the caspase-1 precursor was reduced (Fig. 3C). Consistent with the in vitro experiments (Fig. 2C), upstream expression of ASC was not affected by tetracycline (Fig. 3C). These results indicate that tetracycline reduces silica-induced caspase-1 dependent cytokine production in the lungs.

### Tetracycline ameliorates lung injury in acute silicosis

**Fig. 4:**
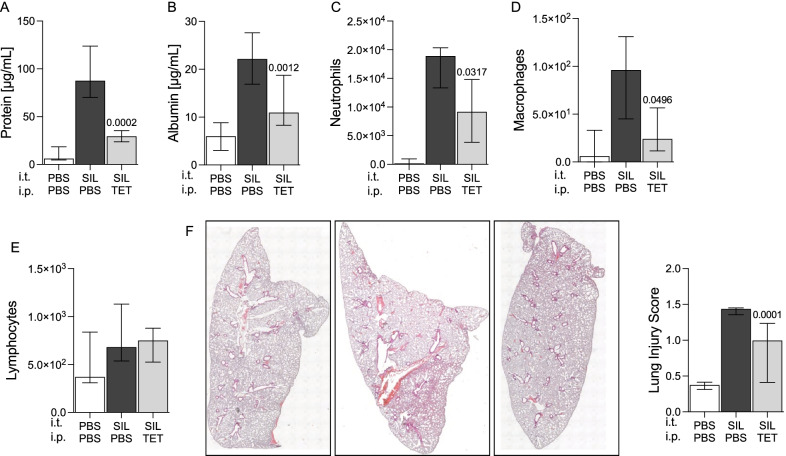
Tetracycline reduces lung injury in silica exposed mice. C57BL/6 J mice were challenged with silica (SIL) and treated with tetracycline (TET) as described in Fig. 3. Total protein (**A**), albumin (**B**), neutrophils (**C**), macrophages (**D**) and lymphocytes (**E**) were quantified in bronchoalveolar lavage fluid by BCA, ELISA and flow cytometry (n ≥ 6 per group). Lungs were removed at 24 h and stained with H&E. Representative histologic sections are shown (magnification, 20×) and lung injury score was determined by examining 5 sections/lung/animal (n = 4 per group, magnification × 100) (**D**). Median with interquartile range of ≥ 3 independent experiments, (SIL + TET (n = 6) vs. SIL + PBS (n = 6)) (PBS + PBS (n = 6)), Mann–Whitney Test

We next examined whether inhibition of caspase-1 could ameliorate disease related lung injury. Therefore, the accumulation of total protein, albumin and neutrophils, macrophages and lymphocytes in BALF was quantified 24 h after silica administration. Silica challenge induced the accumulation of all injury markers (Fig. 4A–D). Treatment with tetracycline significantly reduced silicoproteinosis, indicated by reduced levels of protein (p = 0.0002) and albumin (p = 0.0012) as well as lower numbers of neutrophils (p = 0.0317) and Macrophages (0.0496) in the BALF in comparison to PBS controls. The number of lymphocytes was not affected by the treatment with tetracycline (Fig. 4E). Consistent with reduced neutrophil, macrophage and protein levels in the BALF, the histology of silica exposed lungs revealed that treatment with tetracycline significantly decreases lung injury (Fig. 4F). This series of findings demonstrates that tetracycline can reduce the severity of lung injury in acute silicosis.

### Tetracycline treatment reduces silica-induced chronic lung pathology

**Fig. 5:**
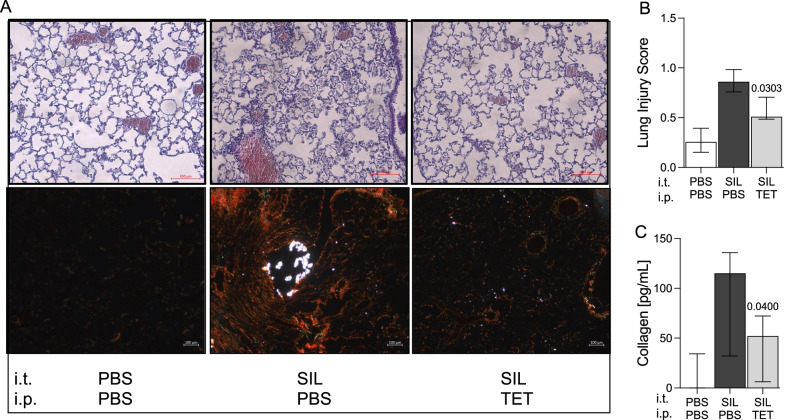
Tetracycline reduces pulmonary fibrotic remodeling in silica exposed mice. C57BL/6 J mice were challenged with silica (SIL) (0.33 mg/mouse) i.t. on days 0, 7, 14 and 21 and treated by i.p. injection of tetracycline (TET) every 24 h for 10d and afterwards 3 times a week. Mice (PBS+PBS (n = 5) SIL+PBS (n = 9) SIL+TET (n = 9)) were sacrificed 12 weeks after instillation. Representative H&E and sirius red stained histologic sections are shown (magnification, 400×) (**A**). Lung injury score was determined by examining 15 sections/lung/animal (n ≥ 5 per group, magnification × 100) (**B**). Pulmonary collagen levels were quantified by SIRCOL collagen assay (**C**). Median with interquartile range of 3 independent experiments, Mann–Whitney Test

Present data reveals that tetracycline effectively reduces silica-induced acute pulmonary inflammation and lung injury (Figs. 3 and 4). Since chronic silicosis results in fibrotic remodeling of the lungs, we investigated the effect of tetracycline in a murine model of long-term silica exposure. Therefore, C57Bl/6 J mice were repetitively i.t. challenged with silica and subsequently treated with tetracycline. Silica exposure led to pronounced lung injury including proteinaceous debris, invasion of leucocytes and alveolar septal thickening (Fig. 5A, B). Treatment with tetracycline significantly ameliorated lung injury (p = 0.0303) (Fig. 5B). Furthermore, substantial collagen deposition was detectable in lungs of silica challenged mice, whereas tetracycline-treated mice possessed significantly reduced collagen levels (p = 0.04) (Fig. 5A, C). In conclusion, these data show protective long-term effects of tetracycline following repeated exposure to silica.

## Discussion

Exposure to silica particles can lead to inflammation of the lung with development of silicosis [23]. Evidence suggests that silica dependent inflammation and lung injury is mediated by excessive production of caspase-1 dependent cytokines and pyroptotic cell death [3–5, 24, 25]. To our knowledge the present report shows for the first time that tetracycline selectively inhibits caspase-1-dependent IL-1β but not NF-ƙB dependent TNF-α production and pyroptosis in response to silica in vitro (Figs. 1 and 2). The results were consistent with tetracycline reducing silica-induced IL-1β and IL-18 production as well as activation of caspase-1 in the lungs (Fig. 3), thereby ameliorating pulmonary inflammation, lung injury (Fig. 4) and subsequent fibrosis (Fig. 5).

Controversies exist whether tetracycline or its derivates selectively inhibit inflammasome-dependent signaling. Lu et al. showed that using minocycline in an oxygen–glucose deprivation / reoxygenation model inhibited both TNF-α (signal 1) and IL-1β (signal 2) in microglia concluding that the effect is unspecific [26]. However, the findings were inconsistent as signal-1 dependent NLRP3 and pro-IL-1β but not pro-caspase-1 production was inhibited by minocycline [26]. Reduced gene expression of NLRP3 and caspase-1 (signal 1) by minocycline has also been described in mouse models of diabetic nephropathy or Huntington disease [10, 27]. Yet, those studies evaluated the effect of minocycline on inflammasome signaling at very late time points [10, 27]. Since IL-1β production is a very early step in the inflammatory response to silica [3, 4], it is likely that secondary effects (such as feedback loops via IL-1R-NF-ƙB) explain the findings of these studies. Current study found no effect of tetracycline on NF-ƙB dependent gene expression of caspase-1 or other inflammasome components in macrophages. Furthermore, tetracycline did not block NLRP3 or ASC production in silica-exposed macrophages, suggesting a tetracycline-mediated blockade of inflammasome-signaling downstream of ASC. Furthermore, there was no effect of tetracycline on pro-IL-1β. Consistent with this, our group recently showed that tetracycline had neither an effect on NF-ƙB dependent gene expression nor on ASC-specking, but rather selectively inhibits activation of caspase-1 in LPS and nigericin activated macrophages [20]. In summary present report suggests that inhibition of IL-1β production and pyroptosis by tetracycline in silica exposed macrophages is mediated via inhibition of caspase-1.

Silica particles cause progressive, inflammasome-associated inflammation and lung injury [3–5]. Increased pulmonary IL-1ß concentrations associated with progressive fibrosis are found in patients with silicosis [28, 29]. Consistent with this, previous studies revealed that IL-1β-, ASC- and NLRP-3-deficient mice were protected from pulmonary inflammation in response to inhaled silica particles [3, 19]. Furthermore, pharmacological intervention by using anti-IL-1β antibody reduced inflammation and lung injury in silica exposed mice [14]. Thus, we questioned whether tetracycline would be a therapeutic option against silica induced inflammation and fibrotic lung remodeling. Therefore, we used a widely accepted murine model featuring i.t. instillation of silica particles [3–5]. For the first time we showed that tetracycline was effective in decreasing silica-induced IL-1β and IL-18 production in silica exposed mice. This was due to tetracycline-mediated inhibition of caspase-1, resulting in significantly reduced lung injury. Comparable effects of tetracycline in reducing pulmonary inflammation have been shown recently in two models of acute lung injury. Here, tetracycline reduced IL-1β and IL-18 levels, pulmonary inflammation and lung injury in mice that were challenged with either LPS or influenza virus [20]. Furthermore, lung injury and inflammation in response to LPS was significantly lower in caspase-1 deficient mice compared to wild-type animals. Of note, tetracycline had no effect on pulmonary inflammation and damage in caspase-1 deficient mice [20].

In summary, current study suggests that tetracycline ameliorates silica-induced inflammation and lung injury via inhibition of caspase-1.

This study has several limitations. First, animal models are limited to mimic the long period of silica exposure (at least 10 years of exposure to low concentrations of silica particles) necessary to induce chronic silicosis in humans [2]. Yet, we evaluated the long-term effects of tetracycline in an established model of chronic silicosis featuring repetitive i.t. instillation of silica particles provoking constitutive pulmonary inflammation [5]. In accordance with others showing amelioration of bleomycin-induced lung fibrosis by doxycycline [13, 14], we found that tetracycline clearly reduced progression of fibrotic lung remodeling and significantly reduced collagen deposition in the lungs of silica challenged mice. This was in line with a chronic silicosis model showing that blocking of IL-1ß reduces pulmonary fibrosis [5].

Second, since BMDM are commonly used to examine silica-induced inflammasome activation [4], we also demonstrate the molecular effects of tetracycline on inflammsasome-caspase-1 pathway in BMDM in vitro. This cannot fully reflect the complex interplay between different cell types orchestrating silica-induced inflammation and lung injury in vivo. Pulmonary inflammation in the context of silicosis is also maintained by non-immune cells and silica exposure leads also to upregulation of NLRP3 inflammasome-caspase-1 activation in lung epithelium [30].

In summary, current study suggests that tetracycline inhibits caspase-1 activation in response to silica and ameliorates silica-induced pulmonary inflammation including IL-1ß production, thereby reducing lung injury and fibrotic lung remodeling.

## Conclusions

Tetracycline reduced caspase-1-dependent production of IL-1β in response to silica in vitro and in vivo. These results were consistent with tetracycline reducing silica-induced pulmonary inflammation and subsequent lung injury and fibrosis in a murine model. Tetracycline and derivates have been shown to provide beneficial immunomodulatory effects in experimental and clinical studies of inflammatory and lung fibrosing diseases including acute lung injury [9, 10, 20]. Since tetracycline is an approved antibiotic drug with a good safety profile and there is a growing number of chemically modified tetracyclines which have been attributed to lack anti-bacterial but retain anti-inflammatory activities [31, 32], tetracycline and its derivates could be repurposed as a protective agent for silica-induced pulmonary inflammation and subsequent disease progression.

## Data Availability

The datasets used and/or analyzed during the current study are available from the corresponding author on reasonable request.

## Abbreviations

ASC: Adapter protein apoptosis-associated speck-like protein containing a CARD domain
ABS: Antibodies
BALF: Bronchoalveolar lavage fluid
BMDM: Bone marrow derived macrophages
H&E: Hematoxylin and eosin
LPS: Lipopolysaccharides
m-CSF: Mouse macrophage colony-stimulating factor
NF-ƙB: Nuclear factor kappa-light-chain-enhancer of activated B cells
NLR: Nucleotide-binding oligomerization domain–like receptor
NLRP3: NLR family pyrin domain–containing 3
PAMP: Pathogen-associated molecular pattern
PRR: Pattern-recognition receptors
TLR: Toll-like receptor
